# miR-4324 inhibits ovarian cancer progression by targeting FEN1

**DOI:** 10.1186/s13048-022-00959-5

**Published:** 2022-03-04

**Authors:** Haixia Wu, Youliang Yan, Jialin Yuan, Mengze Luo, Yingjian Wang

**Affiliations:** 1 Department of Obstetrics and Gynecology, Pinghu Hospital, Health Science Center, Shenzhen University, Shenzhen, 518116 Guangdong P. R. China; 2 Department of Obstetrics and Gynecology, China-Japan Union Hospital of Jilin University, No. 126, Xiantai Avenue, Changchun, 130000 Jilin, P. R. China

**Keywords:** miR-4324, FEN1, Ovarian cancer, Proliferation, Migration, Apoptosis

## Abstract

**Background:**

Ovarian cancer is one of the most lethal malignancies, with a 1.9% mortality rate worldwide. The dysregulation of the *FEN1* gene and miR-4324 has been associated with cancer progression. However, the relationship between miR-4324 and-FEN1 requires further investigation.

**Methods:**

miR-4324 and FEN1 expressions in ovarian cancer tissues and cell lines were measured via RT-qPCR. The interaction between miR-4324 and FEN1 was assessed using luciferase and RNA pull-down assays. The effects of miR-4324 and FEN1 on cell proliferation, adhesion and apoptosis were determined by CCK-8, BrdU, colony formation, cell adhesion, Caspase-3 and western blot assays in ovarian cancer cell lines CaOV3 and OVCAR3, respectively.

**Results:**

The results showed that miR-4324 expression was significantly decreased and FEN1 expression was enhanced in ovarian cancer tissues and cell lines. miR-4324 inhibitor promoted cell proliferation, adhesion and migration, and prevented apoptosis. Furthermore, the downregulation of FEN1 inhibited ovarian cancer cell growth and increased apoptosis. miR-4324 inhibited FEN1 expression and repressed ovarian cancer progression.

**Conclusion:**

Our study found that miR-4324 inhibited FEN1 expression, suppressed cell growth, and increased apoptosis in ovarian cancer cells. Therefore, we identified miR-4324 and FEN1 as potential therapeutic targets for ovarian cancer treatment.

**Supplementary Information:**

The online version contains supplementary material available at 10.1186/s13048-022-00959-5.

## Background

Ovarian cancer is one of the most lethal malignancies, with a worldwide mortality rate of 1.9% [1]. Recent statistics reveal that the mortality rate has increased in China, where 25,000 women diagnosed with ovarian cancer died in 2021 [2]. Although advances in treatments for ovarian cancer such as surgery, chemotherapy, and radiotherapy have improved outcomes for patients, further work in this area is required [3]. Therefore, it is important to elucidate the molecular mechanisms underlying the development of ovarian cancer.

MicroRNAs (miRNAs) contain 20–24 nucleotides and they can regulate the gene expression by binding to the 3′ untranslated region (3′-UTR) of the target mRNAs [4]. miRNAs can serve as tumor suppressors or oncogenes in multiple tumor types [5, 6]. According to the GSE119055 data series, we found that miR-4324 was the second most downregulated miRNA in ovarian cancer. In addition, several studies have indicated that miR-4324 is a tumor suppressor in various cancers, including colorectal cancer, esophageal squamous cell carcinoma (ESCC), and bladder cancer [7–9]. miR-4324 may suppress cell proliferation, migration, and invasion in ovarian cancer, thereby becoming an excellent therapeutic target [7–9]. However, the specific function of miR-4324 in the progression of ovarian cancer needs to be investigated.

miRDB.org was used to predict mRNA targets of miR-4324, and differentially expressed genes (DEGs) of significance were identified using the GEPIA database (http://gepia2.cancer-pku.cn/). The flap structure-specific endonuclease 1 (*FEN1*) was identified as a gene of interest through this approach. The *FEN1* gene is located on chromosome 11q12.2 and consists of two exons [10]. FEN1 is a member of the XPG/RAD2 endonuclease family and is one of ten proteins essential for cell-free DNA replication [10]. Increasing evidence suggests that FEN1 promotes cancer development by promoting cell proliferation, migration, and invasion and decreasing apoptosis. This has been observed in cervical cancer, breast cancer, and non-small cell lung cancer (NSCLC) [11–13]. A study revealed that miR-134-3p, a FEN1 inhibitor, considerably decreased cell proliferation, migration, and invasion and increased apoptosis in human ovarian cancer [14]. However, the function of miR-4324 and FEN1 in ovarian cancer remains unclear.

In this study, we sought to determine the functions of miR-4324 and FEN1 in ovarian cancer. We hypothesized that miR-4324 could attenuate ovarian cancer progression by suppressing FEN1 expression. We identified miR-4324 and FEN1 as potential therapeutic targets for ovarian cancer treatment.

## Materials and methods

### Tissue specimens, cell lines, and cell transfection

Samples of ovarian cancer tissues and adjacent normal tissues (> 3 cm away from the edge of carcinoma) were collected from 40 patients who underwent surgery in our hospital. Each tissue sample was verified as cancerous or non-cancerous via histopathology. All patients provided informed consent prior to the study. This study was approved by the ethics committee of our hospital. A non-cancerous, human ovarian epithelial cell line (HOSEpiC) and multiple ovarian cancer cell lines (SKOV3, CaOV3, and OVCAR3) were provided by ATCC (Manassas, VA, USA). Cells were passaged in RPMI-1640 medium (Gibco, USA) and supplemented with 10% FBS (Gibco, USA) at 37 °C with 5% CO_2_. The miR-4324 (miR-4324 inhibitor), siRNA-FEN1, miR-4324 inhibitor negative controls (inhibitor-NC) and si-FEN1-NC (collectively called NC) were purchased from GenePharm (Shanghai, China). These were transfected into CaOV3 and OVCAR3 cells using Lipofectamine 3000 (Invitrogen, USA) and incubated for 48 h before subsequent experiments. The sequences used in this study are shown in Supplementary Table 1.

### RNA isolation and RT-qPCR analysis

TRIzol reagent (Invitrogen, USA) was used for total mRNA isolation from the ovarian cancer tissues and cell lines. PrimeScript First Strand cDNA Synthesis kit (Cat#: #6110A, Takara, China) was used for cDNA synthesis, and SYBR Premix Ex Taq (Cat#: #RR420A Takara, China) was used for RT-qPCR. In addition, the miRNeasy mini kit (Cat#: #217004, Qiagen, Germany), miScript II RT Kit (Cat#: #218161, QIAGEN, USA), and miScript SYBR Green PCR Kit (Cat#: #218075, QIAGEN, USA) were used for miRNA isolation, cDNA synthesis, and RT-qPCR, respectively, for ovarian cancer tissue and cell lines. Results regarding FEN1 expression and miR-4324 levels were normalized using the 2-^ΔΔCt^ method with GAPDH and U6 as controls, respectively. All primer sequences are listed in Table 1.

**Table 1:** The PCR primer sequences in this study

Genes	Primer sequences
miR-4324	Forward:5′--3′ Reverse:5′--3′
FEN1	Forward:5′-AGCCCGTGTATGTCTTTG-3′ Reverse:5′-AGTCAGGTGTCGCATTAG-3′
GAPDH	Forward:5′-TGCCACTCAGAAGACTGTGG-3′ Reverse:5′-TTCAGCTCTGGGATGACCTT-3′
U6	Forward:5′-GTGCTCGCTTCGGCAGCA-3′ Reverse:5′-CAAAATATGGAACGCTTC-3′

### CCK-8 assay

CaOV3 and OVCAR3 cells (5 × 10^3^) were cultured in 96-well plates. Cell viability was measured using a CCK-8 kit (Cat#: K1018; APExBIO, China). The CCK-8 solution was prepared at a 1:10 dilution in cell culture medium. At 0, 24, 48, and 72 h, the media was removed, and 100 μL of CCK-8 solution was added to each well before incubation at 37 °C for 2 h. The OD value at 450 nm was then determined using a microplate reader (Thermo, USA).

### BrdU assay

The BrdU kit (Cat#: 6813, CST, USA) was used to identify cell proliferation. CaOV3 and OVCAR3 cells (5 × 10^3^) were cultured in 96-well plates. The media was removed, and each well was incubated with BrdU antibody for 2 h at 25 °C. Subsequently, the media was changed twice, and the secondary antibody was added into each well. The plate was incubated at 25 °C for 1 h, and the OD value at 450 nm was determined using a microplate reader (Thermo, USA).

### Cell adhesion assay

CaOV3 and OVCAR3 cells (2 × 10^4^) were seeded into 96-well plates. Collagen I solution (Sigma, USA) was added to a 96-well plate to detect cell adhesion. At 80% density, the cells were washed and maintained in serum-free DMEM for 8 h. After the cells were dissociated with 10 mM EDTA, 100 μL cell suspension was added to the cell adhesion plate and maintained at 37 °C for 30 min and 60 min, respectively. Subsequently, 10 μL of MTT substrate was added to each well and incubated for 2 h at 30 °C. Finally, 100 μL DMSO was used to lyse the cells, and the OD value at 570 nm was determined using a microplate reader (Thermo, USA).

### Apoptosis assay

Apoptosis in both cell lines was detected using a caspase-3 activity assay kit (Cat# 5723, CST, USA) CaOV3 and OVCAR3 cells (5 × 10^3^) were cultured in 96-well plates. Cells were harvested, washed twice, and lysed for 10 min. Following this, caspase-3 activity working solution (100 μL/well) was added to the cell lysate and incubated at 37 °C for 2 h. The OD at 405 nm wavelength was determined using a microplate reader (Thermo, USA).

### Luciferase assay

The pmiRGLO vectors with FEN1 3′-UTRs WT and FEN1 3’UTR MUT1 or MUT2 sequences were purchased from GenePharma (Shanghai, China). The CaOV3 and OVCAR3 cells were co-transfected with 0.24 μg pmiRGLO FEN1 3’UTR WT, MUT1, or MUT2 vectors and 40 nM miR-NC or miR-4324 using Lipofectamine 3000. After 72 h of transfection, the Luciferase Assay Kit (Cat#: #16185, Thermo, USA) was used to measure the activity of firefly and renilla luciferase. The results of the firefly luciferase activities were normalized to renilla luciferase.

### RNA pull-down analysis

The RNA pull-down assay was used to detect interactions between miR-4324 and FEN1 in CaOV3 and OVCAR3 cells. The biotin-labeled miR-4324 mutant, miR-4324 mimic, and antisense oligo were purchased from Thermo Fisher (USA). Cell lysate suspensions were incubated with streptavidin beads (Cat#: #88817, Thermo Fisher, USA) conjugated to the biotin-labeled miR-4324 mutant, miR-4324 mimic and antisense oligo at 4 °C overnight. Next, the eluted solution was purified using the RNeasy Mini Kit (Cat#: 74104, QIAGEN, Germany). Finally, the enrichment of FEN1 was determined using RT-qPCR.

### Western blotting analysis

The transfected CaOV3 and OVCAR3 cells were harvested using RIPA buffer. (Cat#: #20–188, Sigma, USA). Equal amounts of protein from each sample were loaded on to 10% SDS-PAGE and transferred to a PVDF membrane. After blocking in TBST containing 5% non-fat milk, membranes were washed three times. The membranes were incubated with anti-FEN1 (1:1000, Cat#: ab153825, Abcam, UK) and anti-GAPDH (1:2000, Cat#: ab9485, Abcam, UK) antibodies overnight at 4 °C. After washing with TBST three times, the membranes were incubated with anti-HRP-Rabbit antibody for 1 h at 25 °C. Protein visualization was conducted using ECL reagents (Bio-Rad, Hercules, CA, USA) and analyzed using Image Lab software (Bio-Rad, USA). Relative FEN1 protein expression was normalized to the GAPDH control.

### Statistical analysis

Data was collected from three independent experiments. Statistical analysis involved the use of a paired Student’s t-test and one-way ANOVA followed by Dunnett’s post-hoc test for the analysis of two groups and multiple groups, respectively. This analysis was performed using GraphPad 8.0 software (GraphPad, USA) and presented as the mean ± SD. Pearson correlation analysis was used to analyze FEN1 and miR-4324 expression in ovarian cancer tissues. Statistical significance was set at *P* < 0.05.

## Results

### miR-4324 expression was repressed in ovarian cancer

**Fig. 1:**
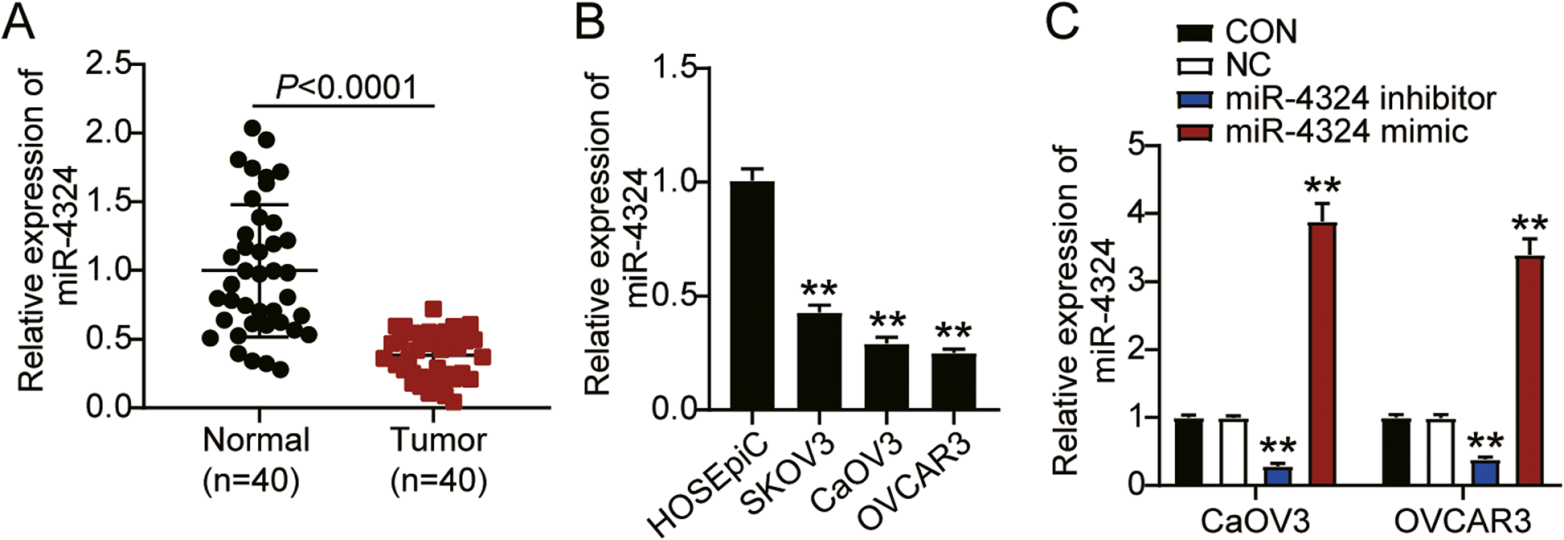
MiR-4324 expression was downregulated in ovarian cancer. **A** RT-qPCR detection of miR-4324 expression in ovarian cancer tissues (*n* = 40) and normal tissues (*n* = 40). **B** Measurement of miR-4324 expression in ovarian cancer cells lines (SKOV3, CaOV3, and OVCAR3) and normal ovarian epithelial cell (HOSEpiC). **C** Measurement of miR-4324 expression in CaOV3 and OVCAR3 cells transfected with NC and miR-4324 inhibitor by RT-qPCR. *, *P* < 0.05; **, *P* < 0.001. NC, negative control; inhibitor, miR-4324 inhibitor

To investigate the role of miR-4324 in ovarian cancer, we first measured miR-4324 expression in ovarian cancer tissues and found that it was significantly lower in tumor tissues than in normal tissues (Fig. 1A). In addition, miR-4324 expression was evidently repressed in ovarian cancer cell lines (SKOV3, CaOV3, and OVCAR3) when compared to normal ovarian epithelial cells (HOSEpiC). The CaOV3 and OVCAR3 cells displayed the lowest miR-4324 expression, and thus these cell lines were selected for use in subsequent experiments (Fig. 1B). We then transfected the miR-4324 inhibitor and inhibitor-NC into CaOV3 and OVCAR3 cells. It was found that miR-4324 expression was downregulated by more than 50% in cells transfected with the inhibitor, compared to the control cells. This finding was consistent across both cell lines (Fig. 1C).

### Downregulation of miR-4324 expression facilitated tumorigenesis in ovarian cancer

**Fig. 2:**
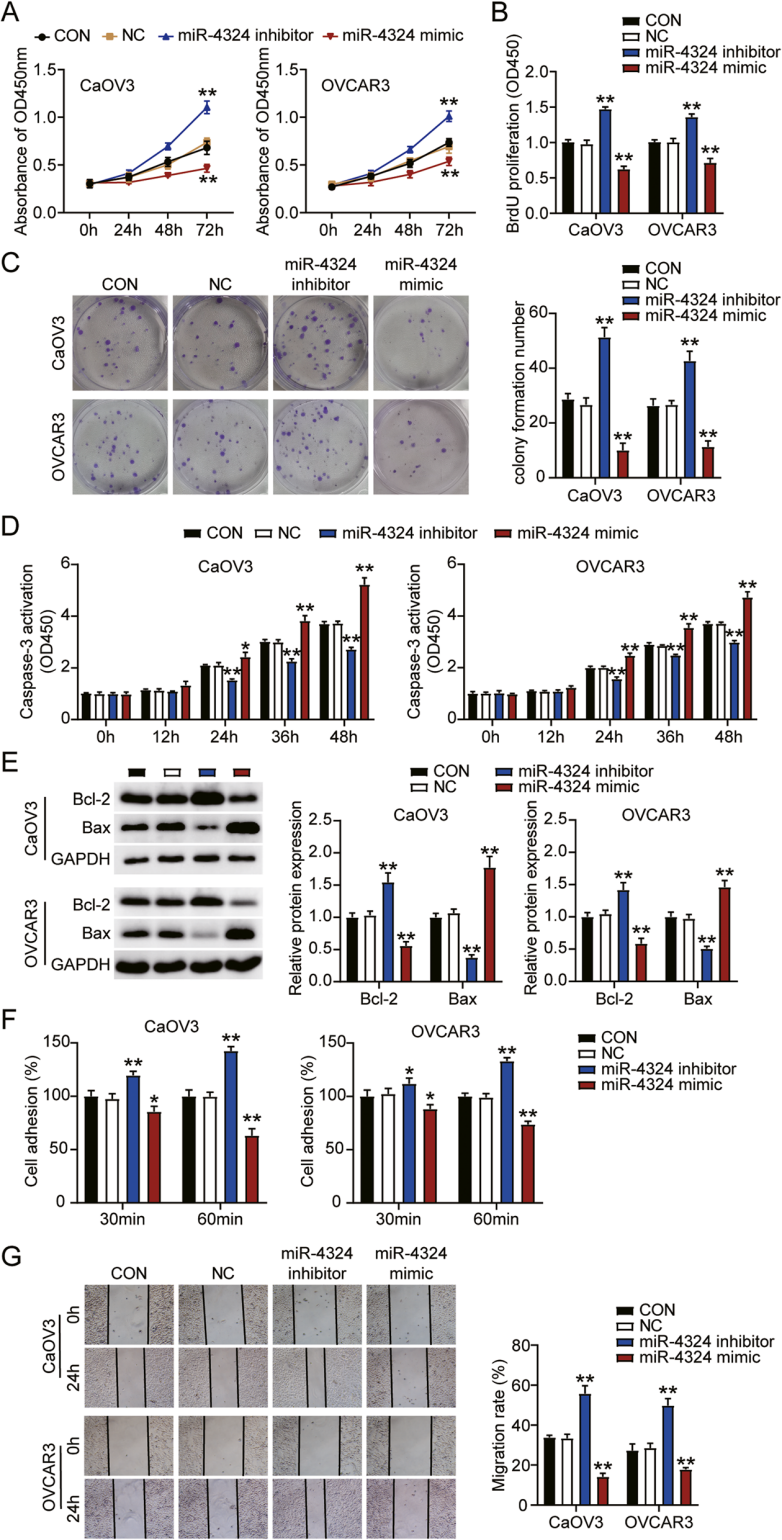
Downregulation of miR-4324 facilitated tumorigenesis in ovarian cancer. **A** Cell viability was detected in CaOV3 and OVCAR3 cells transfected with miR-4324 inhibitor and mimic by CCK-8 assay. **B** Cell proliferation was detected in CaOV3 and OVCAR3 cells transfected with miR-4324 inhibitor and mimic by BrdU assay. **C** Colony formation number was detected in CaOV3 and OVCAR3 cells transfected with miR-4324 inhibitor and mimic by colony formation assay. **D** Cell apoptosis level was determined in CaOV3 and OVCAR3 cells transfected with miR-4324 inhibitor and mimic by Caspase 3 kit. **E** Bax and Bcl-2 protein levels were detected in CaOV3 and OVCAR3 cells transfected with miR-4324 inhibitor and mimic by western blot assay. **F** Cell adhesion was detected in CaOV3 and OVCAR3 cells transfected with miR-4324 inhibitor and mimic. **G** Cell migration rate was detected in CaOV3 and OVCAR3 cells transfected with miR-4324 inhibitor and mimic by wound healing assay. *, *P* < 0.05; **, *P* < 0.001. NC, negative control; inhibitor, miR-4324 inhibitor; mimic, miR-4324 mimic

Next, we performed a series of experiments to confirm cell phenotype following miR-4324 inhibitor treatment. Cell viability significantly increased in the inhibitor groups and decreased in the mimic groups when compared with the control group in both cell lines (Fig. 2A). In addition, the inhibitor groups exhibited approximately a 1.5-fold increase in cell proliferation when compared with the control cells in both cell types, whereas the mimic groups exhibited 40% decrease in cell proliferation (Fig. 2B). Colony formation analysis revealed that miR-4324 inhibitor increased colony formation number, whereas miR-4324 mimic decreased colony formation number (Fig. 2C).Furthermore, the inhibitor groups had decreased rates of apoptosis compared with control cells in both cell lines after 24 h, whereas the mimic groups had increased rates of apoptosis (Fig. 2D). Similarly, western blot analysis showed that compared with the control groups, Bcl-2 increased and Bax decreased in the inhibitor groups, while Bcl-2 decreased and Bax increased in the mimic groups (Fig. 2E). Moreover, the inhibitor groups exhibited significantly increased cell adhesion compared with control cells, whereas the mimics group exhibited significantly decreased cell adhesion. This finding was consistent across both cell lines (Fig. 2F). The wound healing assay revealed an enhanced migration level in inhibitor groups, and a decreased migration level in mimic groups (Fig. 2G).Overall, these results demonstrate that miR-4324 suppresses cell proliferation and adhesion, while significantly increasing apoptosis in ovarian cancer.

### miR-4324 targets and inhibits expression of FEN1

**Fig. 3:**
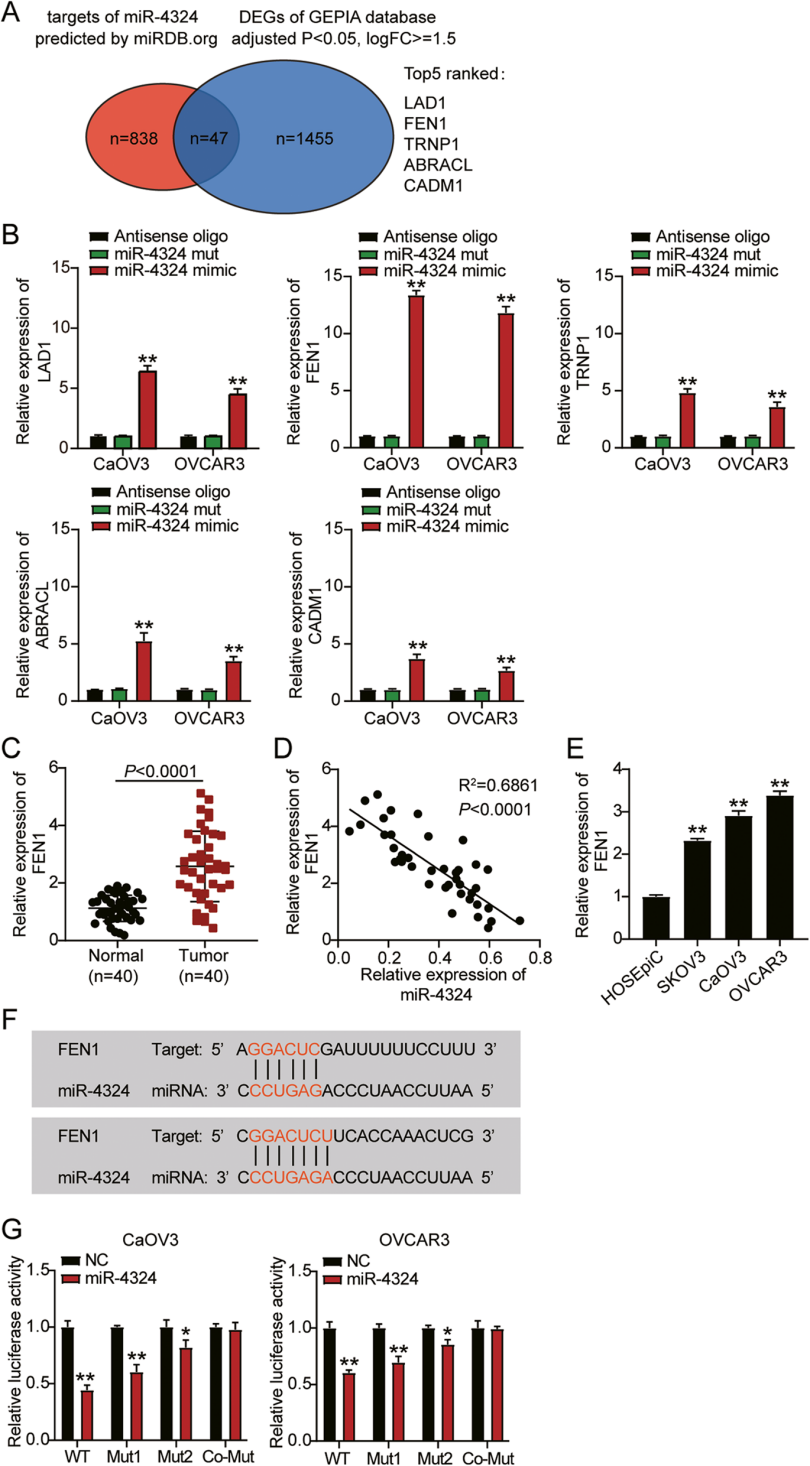
MiR-4324 targeting FEN1 and inhibited the expression of FEN1. **A** A Venn diagram showing the intersection between the predicted downstream genes of miR-4324 by miRDB.org database and the significantly DEGs from GEPIA database. **B** RT-qPCR detection of expression of LAD1, FEN1, TRNP1, ABRACL and CADM1 in CaOV3 and OVCAR3 cells transfected with Antisense oligo, Bio-miR-4324 mut or Bio-miR-4324 mimic. **C** RT-qPCR detection of expression of FEN1 in the ovarian cancer tissues and normal tissues. **D** Correlation analysis between the miR-4324 expression and FEN1 expression in the ovarian tumor tissues. **E** Measurement of FEN1 expression in ovarian cancer cells lines (SKOV3, CaOV3, and OVCAR3) and normal ovarian epithelial cell (HOSEpiC). **F** Bioinformatics analysis showed two binding sites sequence of miR-4324 and FEN1 3′-UTR. **G** Dual luciferase assay was performed in cells co-transfected with plasmids FEN1-WT or FEN1-MUT1 or MUT2 and miR-NC or miR-4324 mimic in CaOV3 and OVCAR3 cells. *, *P* < 0.05; **, *P* < 0.001. WT, wild-type; MUT, mutant; NC, negative control

We identified 29 differentially expressed miRNAs (DE-miRs) from the GSE119055 data series. DE-miRs were defined as miRNAs with an adjusted *p* value of < 0.05 and |logFC| > =1.5 (Supplementary Table 2). Among the 29 DE-miRs, miR-383-5p was the most significantly downregulated in ovarian cancer, and has been the subject of multiple studies [15, 16]. miR-4324, ranked as the second most significantly downregulated miRNA in ovarian cancer, has been identified as a tumor suppressor in colorectal cancer [7] and bladder cancer [9], but not in ovarian cancer. Thus, we selected miR-4324 as our gene of interest. We identified the predicted targets of miR-4324 using miRDB.org and DEGs of significance using the GEPIA database (http://gepia2.cancer-pku.cn/), and identified 47 common genes (Fig. 3A). The enrichment degree of these 5 genes on biotin-labeled miR-4324 mimic was identified by RNA pull-down, and the expression level of FEN1 in miR-4324 mimic group increased the most compared with other genes (Fig. 3B). FEN1 has been extensively studied and identified as a tumor promoter in breast cancer [12, 17] and hepatocellular cancer [18]. Thus, we speculated that FEN1 might be a tumor promoter in ovarian cancer. FEN1 expression levels in ovarian cancer tissues were approximately double those of normal tissues (Fig. 3C). Moreover, we observed a negative correlation between miR-4324 and FEN1 expression in ovarian tumor tissues (Fig. 3D). FEN1 expression was also significantly higher in ovarian cancer cell lines (SKOV3, CaOV3, and OVCAR3) than in the non-cancerous cell line (HOSEpiC) (Fig. 3E). miRdb analysis revealed two possible sites where FEN1 and miR-4324 might bind to each other (Fig. 3F). Thus, we transfected pmiRGLO vectors with wild-type (WT) FEN1 3′-UTRs and MUT1 or MUT2 sequences, and miR-4324-NC (negative control) or miR-4324-mimic into CaOV3 and OVCAR3 cells. Luciferase activity in the WT FEN1 3-UTR and miR-4324-mimic treated cells was significantly downregulated. Luciferase activity partially decreased in cells treated with one of the FEN1 3-UTR MUT vectors, suggesting that miR-4324 targeted both sites (Fig. 3G).

### miR-4324 targets FEN1 attenuating the progression of ovarian cancer

**Fig. 4:**
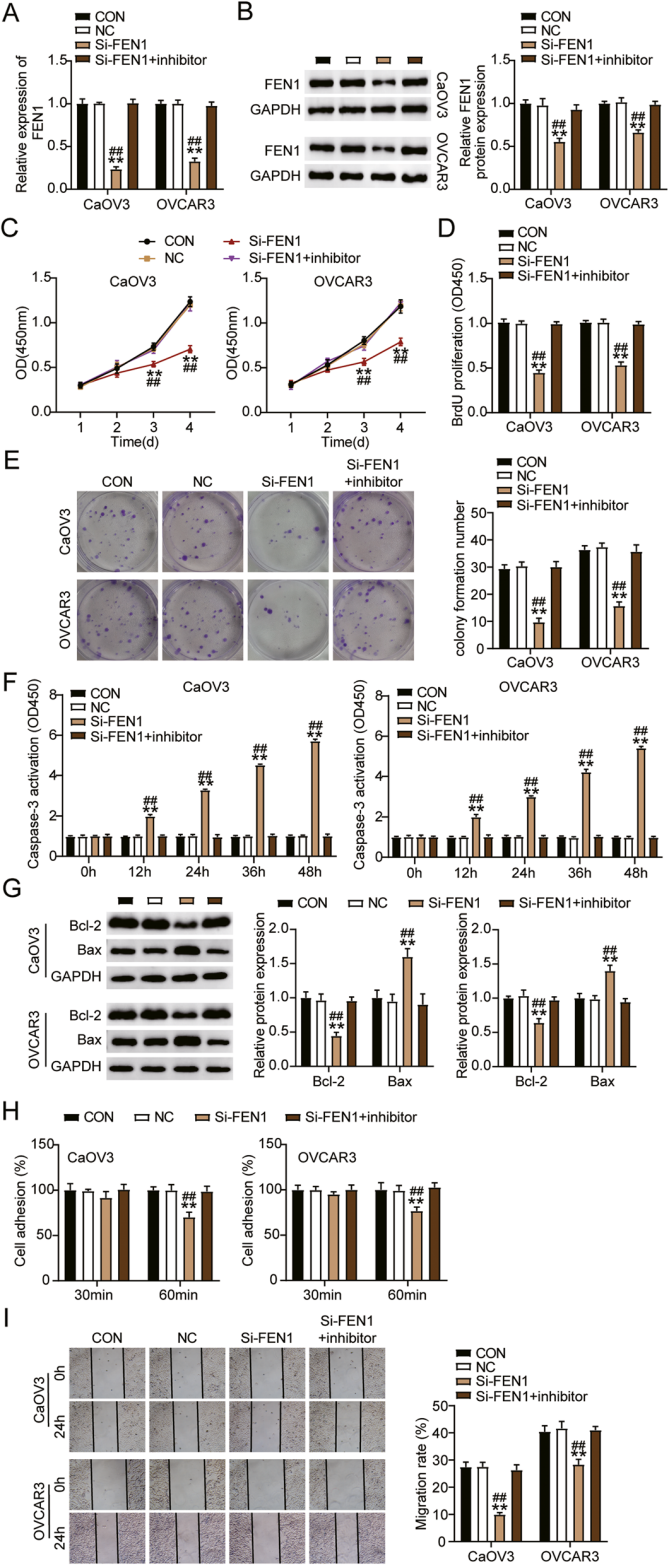
MiR-4324 targeting FEN1 attenuated the progression of ovarian cancer. **A** Measurement of FEN1 expression in CaOV3 and OVCAR3 cells transfected with NC, Si- FEN1, and Si- FEN1+ inhibitor by RT-qPCR. **B** Measurement of FEN1 protein expression in CaOV3 and OVCAR3 cells transfected with NC, Si- FEN1, and Si- FEN1+ inhibitor by western blot. **C** Cell viability was detected in CaOV3 and OVCAR3 cells transfected with NC, Si- FEN1, and Si- FEN1+ inhibitor by CCK-8 assay. **D** Cell proliferation was detected in CaOV3 and OVCAR3 cells transfected with NC, Si- FEN1, and Si- FEN1+ inhibitor by BrdU assay. **E** Colony formation number was detected in CaOV3 and OVCAR3 cells transfected with NC, Si- FEN1, and Si- FEN1+ inhibitor by colony formation assay. **F** Cell apoptosis level was determined in CaOV3 and OVCAR3 cells transfected with NC, Si- FEN1, and Si- FEN1+ inhibitor by Caspase 3 kit. **G** Bax and Bcl-2 protein levels were detected in CaOV3 and OVCAR3 cells transfected with NC, Si- FEN1, and Si- FEN1+ inhibitor by western blot assay. **H** Cell adhesion was detected in CaOV3 and OVCAR3 cells transfected with NC, Si- FEN1, and Si- FEN1+ inhibitor. **I** Cell migration rate was detected in CaOV3 and OVCAR3 cells transfected with NC, Si- FEN1, and Si- FEN1+ inhibitor by wound healing assay. *, *P* < 0.05; **, *P* < 0.001. NC, negative control; Si- FEN1, SiRNA-FEN1; inhibitor, miR-4324 inhibitor; Si- FEN1+ inhibitor, SiRNA-FEN1 + miR-4324 inhibitor

To determine the function of the miR-4324-FEN1 axis, we transfected FEN1 siRNA and miR-4324-inhibitor into CaOV3 and OVCAR3 cells. The siFEN1 groups exhibited 60% decreased FEN1 expression compared with control cells, whereas the siFEN1 + inhibitor groups had similar levels of FEN1 expression to control cells across cell lines (Fig. 4A). FEN1 protein level in the siFEN1 group was approximately 50% lower than that of the control cells in both cell lines. However, FEN1 protein expression in the siFEN1 + inhibitor groups was comparable to that of the control group (Fig. 4B). Next, we found that the siFEN1 groups presented significantly increased cell viability compared to control cells, a result not observed in the siFEN1 + inhibitor groups (Fig. 4C). In addition, the Si-FEN1 groups showed a decreased cell proliferation and colony formation number compared with control cells, however, the Si-FEN1+ inhibitor groups counteracted this effect (Fig. 4D and E). Furthermore, the siFEN1 groups exhibited significantly increased cell apoptosis compared with control cells after 12 h, whereas the siFEN1 + inhibitor groups exhibited no difference (Fig. 4F). The expression of Bcl-2 protein in si-FEN1 groups was lower than that in control group, while Bax was higher than that in control group. Besides, the expression of Bax and Bcl-2 protein in Si-FEN1+ inhibitor groups was similar to that in control group (Fig. 4G). The siFEN1 groups exhibited approximately 30% less cell adhesion compared with control cells after 60 min of treatment, whereas the siFEN1 + inhibitor groups had similar cell adhesion levels to the control cells (Fig. 4H). Wound healing analysis revealed that cell migration was significantly reduced in the Si-FEN1 groups compared with the control group, while there was no significant difference in cell migration in the Si-FEN1+ inhibitor groups (Fig. 4I). Overall, these results indicated that miR-4324 targets FEN1, thereby impairing the development of ovarian cancer.

## Discussion

This study revealed that miR-4324 expression was significantly downregulated and FEN1 expression upregulated in ovarian cancer tissues and cell lines. miR-4324 expression was negatively correlated with FEN1 expression in ovarian cancer tissues. miR-4324 inhibited FEN1 expression and suppressed cell growth, while increasing apoptosis in CaOV3 and OVCAR3 ovarian cancer cells.

Recent evidence has revealed that miR-4324 inhibits cellular growth and metastasis in multiple cancer types [7–9]. Li et al. reported that miR-4324 functions as a tumor suppressor in colorectal cancer by targeting HOXB2. The upregulation of miR-4324 significantly attenuates cell proliferation, migration, and invasion [7]. Zhou et al. demonstrated that miR-4324 directly targets FAK and suppresses ESCC cell growth, and the downregulation of miR-4324 promotes the epithelial-to-mesenchymal transition (EMT) of ESCC cells by upregulating FAK [8]. Ge et al. revealed that the overexpression of miR-4324 in bladder cancer cells drastically represses cell proliferation and metastasis. Reportedly, increased miR-4324 expression increases sensitivity to the chemotherapeutic drug, doxorubicin, by inhibiting RACGAP1 expression [9]. In our study, we demonstrated that miR-4324 expression was significantly repressed in both ovarian cancer tissues and cells. The addition of an miR-4324 inhibitor facilitated cell growth and inhibited apoptosis in ovarian cancer cell lines CaOV3 and OVCAR3. These findings correspond with the findings of previous studies, which report that miR-4324 acts as a tumor suppressor. Moreover, this study further demonstrated that miR-4324 targeted FEN1 and repressed cell growth and adhesion in ovarian cancer. In conclusion, this study revealed that miR-4324 plays a role in preventing ovarian cancer.

An increasing number of studies have suggested that FEN1 plays an extremely important role in the pathogenesis in multiple cancers, including breast cancer, NSCLC, and gastric cancer [13, 17, 19]. Zeng et al. demonstrated that FEN1 mediates miR-200a methylation and promotes breast cancer cell growth via MET and EGFR signaling [17]. Additionally, the overexpression of FEN1 increases cell proliferation and is associated with poor prognosis in NSCLC [13]. Moreover, Wang et al. reported that FEN1 is a promising candidate biomarker for gastric cancer, and that it promotes cell proliferation and inhibits cell apoptosis [19]. In the context of ovarian cancer, a previous study revealed that FEN1 could be a key biomarker of ovarian cancer owing to the increased expression of FEN1 mRNA and protein [20]. Zhao et al. found that the *FEN1* gene is downstream of miR-134-3p, and that the upregulation of FEN1 reversed the effects of the miR-134-3p mimic on cell proliferation, migration, and invasion in ovarian cancer cell lines SKOV-3 and OVCAR-3 [14]. Consistent with the study of Zhao [14], our study revealed that FEN1 expression was significantly increased in ovarian tissues and cell lines, and the knockdown of FEN1 reduced cell proliferation and adhesion but promoted cell apoptosis. Furthermore, we found, for the first time, that the effect of FEN1 on ovarian cancer cells was inhibited by miR-4324. It is likely that miR-4324/FEN1 interaction could be a novel molecular target for ovarian cancer treatment in the future.

Evidence suggests that NF-κB/p65 directly binds to the FEN1 promoter and enhances FEN1 transcription, which contributes to the AKT signaling pathway to drug resistance in cancer cells [21]. The pathways involved in the miR-4324-FEN1 axis in ovarian cancer remain unclear. Further studies investigating the specific signaling pathways involved in this axis are warranted and will be performed in the future. In addition, the confirmation of the miR-4324-FEN1 axis in ovarian cancer is needed.

In summary, this study revealed that miR-4324 suppressed ovarian cancer cell proliferation and adhesion by inhibiting FEN1 expression. This finding validated the role of the miR-4324-FEN1 axis in ovarian cancer genesis, a discovery which has allowed both miR-4324 and FEN1 to be identified as possible future targets for ovarian cancer therapy.

## Supplementary Information

**Additional file 1: Supplementary Table 1.** The sequences of miR-4324 inhibitor, miR-4324 mimic, Si-FEN1 and negative control.

**Additional file 2: Supplementary Table 2.** The significantly differentially expressed miRNAs of GSE119055 data series with the criteria of adjusted *P* < 0.05 and |logFC| > =1.5.

## Data Availability

The datasets used and analyzed during the current study are available from the corresponding author on reasonable request.
