# Supplementary material for: miR-4324 inhibits ovarian cancer progression by targeting FEN1
Source: J Ovarian Res. 2022 Mar 4;15:32. doi: 10.1186/s13048-022-00959-5 (PMC8896303; doi:10.1186/s13048-022-00959-5)
Supplement: Supplementary file 1 — Additional file 1: Supplementary Table 1. The sequences of miR-4324 inhibitor, miR-4324 mimic, Si-FEN1 and negative control. [file 13048_2022_959_MOESM1_ESM.docx]

Supplementary Table 1. The sequences of miR-4324 inhibitor, miR-4324 mimic, Si-FEN1 and negative control.

| Characteristic | Sequence |
| --- | --- |
| miR-4324 inhibitor | 5’-UUGUUAAGGGUCUCAGCUCCAGG-3’ |
| miR-4324 mimic | 5’-CCCUGAGACCCUAACCUUAA-3’ |
| Si-FEN1 | 5’-GGGTCAAGAGGCTGAGTAA-3’ (sense) |
|  | 5’-UUACUCAGCCUCUUGACCCdTd-T-3’ (antisense) |
| NC | 5’-TTCTCCGAACGTGTCACGT-3’ |
