# Supplementary material for: miR-4324 inhibits ovarian cancer progression by targeting FEN1
Source: J Ovarian Res. 2022 Mar 4;15:32. doi: 10.1186/s13048-022-00959-5 (PMC8896303; doi:10.1186/s13048-022-00959-5)
Supplement: Supplementary file 2 — Additional file 2: Supplementary Table 2. The significantly differentially expressed miRNAs of GSE119055 data series with the criteria of adjusted P < 0.05 and |logFC| > =1.5. [file 13048_2022_959_MOESM2_ESM.docx]

Supplementary Table 2. The significantly differentially expressed miRNAs of GSE119055 data series with the criteria of adjusted P<0.05 and |logFC|>=1.5.

| ID | Adjusted P Value | logFC^*^ | Sequence | miRNA ID |
| --- | --- | --- | --- | --- |
| 20501252 | 0.000185 | -7.6959288 | AGAUCAGAAGGUGAUUGUGGCU | hsa-miR-383-5p |
| 20517704 | 0.023007 | -4.7264139 | CCCUGAGACCCUAACCUUAA | **hsa-miR-4324** |
| 20503879 | 0.011436 | -4.2205063 | AUCCUUGCUAUCUGGGUGCUA | hsa-miR-502-5p |
| 20501202 | 0.02311 | -4.0582823 | AACACACCUAUUCAAGGAUUCA | hsa-miR-362-3p |
| 20500182 | 0.031628 | -3.785004 | CAAGCUCGCUUCUAUGGGUCUG | hsa-miR-99a-3p |
| 20504432 | 0.020382 | -3.4805284 | ACCUCCUGUGUGCAUGGAUUA | hsa-miR-660-3p |
| 20517755 | 0.023007 | -3.4405819 | GCACCCAGGCAAGGAUUCUG | hsa-miR-500b-3p |
| 20506838 | 0.038476 | -3.1741603 | ACCCGUCCCGUUCGUCCCCGGA | hsa-miR-1247-5p |
| 20501160 | 0.011436 | -3.1266025 | UAGCACCAUUUGAAAUCGGUUA | hsa-miR-29c-3p |
| 20500185 | 0.00322 | -2.8869211 | CAGUUAUCACAGUGCUGAUGCU | hsa-miR-101-5p |
| 20503878 | 0.013204 | -2.6723958 | AAUGCACCCGGGCAAGGAUUCU | hsa-miR-501-3p |
| 20503908 | 0.046462 | -2.5683031 | CCUCCCACACCCAAGGCUUGCA | hsa-miR-532-3p |
| 20501278 | 0.033793 | -2.5381184 | CUGGCCCUCUCUGCCCUUCCGU | hsa-miR-328-3p |
| 20504431 | 0.038476 | -2.48161 | UACCCAUUGCAUAUCGGAGUUG | hsa-miR-660-5p |
| 20518907 | 0.023088 | -2.4647022 | UGAGGGAGUAGGAUGUAUGGUU | hsa-miR-4510 |
| 20500155 | 0.007958 | -2.431247 | CCUGUUCUCCAUUACUUGGCUC | hsa-miR-26b-3p |
| 20506822 | 0.011436 | -2.4305664 | UGUGAGGUUGGCAUUGUUGUCU | hsa-miR-1294 |
| 20501309 | 0.035515 | -2.4123718 | UUUGGUCCCCUUCAACCAGCUA | hsa-miR-133b |
| 20500154 | 0.038969 | -2.3348249 | UUCAAGUAAUUCAGGAUAGGU | hsa-miR-26b-5p |
| 20511563 | 0.019308 | -2.304032 | GCUGGUGCAAAAGUAAUGGCGG | hsa-miR-548q |
| 20503880 | 0.047883 | -2.2354918 | AAUGCACCUGGGCAAGGAUUCA | hsa-miR-502-3p |
| 20503876 | 0.038476 | -2.2261676 | AUGCACCUGGGCAAGGAUUCUG | hsa-miR-500a-3p |
| 20500181 | 0.023812 | -2.1277025 | AACCCGUAGAUCCGAUCUUGUG | hsa-miR-99a-5p |
| 20515627 | 0.023088 | -1.9661418 | AAAGCUGGGUUGAGAAGG | hsa-miR-320e |
| 20518924 | 0.023812 | -1.8561916 | UGAGACAGGCUUAUGCUGCUAU | hsa-miR-4524a-3p |
| 20518838 | 0.023088 | -1.6130704 | AGAGGUAGGUGUGGAAGAA | hsa-miR-4458 |
| 20504566 | 0.02311 | -1.5080624 | CUCCGUUUGCCUGUUUCGCUG | hsa-miR-1468-5p |
| 20500142 | 0.010795 | 3.523981 | CAACACCAGUCGAUGGGCUGU | hsa-miR-21-3p |
| 20500455 | 0.023007 | 5.2186374 | UCGUGUCUUGUGUUGCAGCCGG | hsa-miR-187-3p |

^*^: fold change.
